# Caspases: structural and molecular mechanisms and functions in cell death, innate immunity, and disease

**DOI:** 10.1038/s41421-025-00791-3

**Published:** 2025-05-05

**Authors:** Eswar Kumar Nadendla, Rebecca E. Tweedell, Gary Kasof, Thirumala-Devi Kanneganti

**Affiliations:** 1 Department of Immunology, St. Jude Children’s Research Hospital, Memphis, TN USA; 2 Cell Signaling Technology, Danvers, MA USA

**Keywords:** Structural biology, Cell biology

## Abstract

Caspases are critical regulators of cell death, development, innate immunity, host defense, and disease. Upon detection of pathogens, damage-associated molecular patterns, cytokines, or other homeostatic disruptions, innate immune sensors, such as NLRs, activate caspases to initiate distinct regulated cell death pathways, including non-lytic (apoptosis) and innate immune lytic (pyroptosis and PANoptosis) pathways. These cell death pathways are driven by specific caspases and distinguished by their unique molecular mechanisms, supramolecular complexes, and enzymatic properties. Traditionally, caspases are classified as either apoptotic (caspase-2, -3, -6, -7, -8, -9, and -10) or inflammatory (caspase-1, -4, -5, and -11). However, extensive data from the past decades have shown that apoptotic caspases can also drive lytic inflammatory cell death downstream of innate immune sensing and inflammatory responses, such as in the case of caspase-3, -6, -7, and -8. Therefore, more inclusive classification systems based on function, substrate specificity, or the presence of pro-domains have been proposed to better reflect the multifaceted roles of caspases. In this review, we categorize caspases into CARD-, DED-, and short/no pro-domain-containing groups and examine their critical functions in innate immunity and cell death, along with their structural and molecular mechanisms, including active site/exosite properties and substrates. Additionally, we highlight the emerging roles of caspases in cellular homeostasis and therapeutic targeting. Given the clinical relevance of caspases across multiple diseases, improved understanding of these proteins and their structure–function relationships is critical for developing effective treatment strategies.

## Introduction

The innate immune system comprises genetically encoded sensors of pathogen- and damage-associated molecular patterns (PAMPs and DAMPs) and other homeostatic alterations to provide the first line of defense and drive inflammation and cell death to maintain homeostasis^1–3^. Regulated cell death pathways are genetically defined and play central roles in innate immunity^4–7^. Among the well-characterized cell death pathways are non-lytic apoptosis, as well as innate immune lytic pathways, including pyroptosis and PANoptosis^7–12^. Apoptosis has been characterized as a non-lytic, non-inflammatory form of cell death with key roles in organismal development and cellular homeostasis^12,13^. In contrast, the lytic innate immune cell death pathways pyroptosis and PANoptosis are initiated downstream of innate immune sensor activation and result in cellular lysis and the release of DAMPs and other inflammatory factors^8,10,11,14–27^. Among the diverse non-lytic and lytic cell death mechanisms, caspases often act at the core of regulation and execution in all metazoans, including *C. elegans, Drosophila*, mice, and humans^28–30^, to critically regulate inflammation and innate immunity, as well as proliferation, differentiation, and remodeling in higher-order organisms^11,29^ (Table 1). While apoptosis, pyroptosis, and PANoptosis are driven by the activation of caspases, another lytic form of cell death, necroptosis, is driven by the inhibition of a caspase, as discussed in detail below.

**Table 1:** Structures, select interacting partners, and cellular functions of human caspases and their disease associations.

Caspase	PDB entry^a^	Select interacting partner^b^	Cellular function	Disease^c^	Disease-associating mutation^d^	Inhibitor	Reference
CASP1	1IBC,1ICE,1RWK,1RWM,1RWN,1RWO,1RWP,1RWV,1RWW,1RWX,1SC1,1SC3,1SC4,2FQQ,2H48,2H4W,2H4Y,2H51,2H54,2HBQ,2HBR,2HBY,2HBZ,5MMV,5MTK,6PZP,1BMQ,3D6F,3D6H,3D6M,3NS7,6BZ9,6F6R,3E4C,5FNA,8WRA,6VIE,6KN0,8SV1,7KEU	ARID4B,BRCA1,CARD16,FMN2,GAB1,GSDMD,IFI16,IL1B,KIF11,KIF3A,NLRC4,PI4KB,PLCL2,PYCARD,RNF31,VAC14	Pyroptosis,PANoptosis,Metabolism	Cancer (Colorectal, Lung, Prostate, Breast, Hepatocellular, Pancreatic, Ovarian, Gastric), Osteoporosis, Parkinson (late onset), Ataxia-telangiectasia, Multiple sclerosis, Rheumatoid arthritis, Retinitis pigmentosa, Lynch syndrome, Pulmonary hypertension, Cone-Rod dystrophy, Sickle cell, Cardio- and nephrological diseases, Schizophrenia, Familial Mediterranean fever, Asthma, Chronic lymphocytic leukemia	K37Q, V61I, C72F	Minocycline, Pralnacasan, LAX-101, Emricasan, VX-765, Z-VAD, VX-166, Belnacasan	^11,20,262,269,270^
CASP2	3R5J,3R6G,3R6L,3R7B,3R7N,3R7S,1PYO,2P2C,3RJM,6GKF,6GKG,6S9K,6SAD,6Y8B,6Y8D	BBOX1,BID,BIRC2,CASP3,CASP8,CIB1,CRADD,EPN3,HNRNPK,PIDD1,RFXANK,TRAF1,TRAF2,TRAF3	Cell cycle,Autophagy,Genome stability,Tumorigenesis,Tumor suppression,Aging,Apoptosis	Cancer (Colorectal, Lung, Prostate, Breast, Hepatocellular, Pancreatic, Ovarian, Gastric, Head and neck, Brain), Familial Alzheimer disease, Osteoporosis, Ataxia-telangiectasia, Ischemic stroke, Multiple sclerosis, Lynch syndrome, Neurofibromatosis type I, Lung cancer susceptibility, Myeloma, Multiple leukemia, Chronic lymphocytic squamous cell carcinoma	R234G^lb^, R265^lb^	Emricasan, Z-VAD, VX-166	^11,271^
CASP3	1GFW,1NME,1PAU,1RE1,1RHJ,1RHK,1RHM,1RHQ,1RHR,1RHU,2C1E,2C2K,2C2M,2C2O,2CDR,2CJX,2CJY,2CNK,2CNL,2CNN,2CNO,2DKO,2H5I,2H5J,2H65,2XYG,2XYH,2XYP,2XZD,2XZT,2Y0B,3EDQ,3GJQ,3GJR,3GJS,3GJT,3KJF,4DCJ,4DCO,4DCP,5IC4,6BDV,6BFJ,6BFK,6BFL,6BFO,6BG0,6BG1,6BG4,6BGK,6BGQ,6BGR,6BGS,6BH9,6BHA,6CKZ,6CL0,6X8I,6X8K,7RN7,7RN8,7RN9,7RNA,7RNB,7RNC,7RND,7RNE,7RNF,7RNG,7SEO,7USO,7USP,7USQ,1CP3,1NMQ,1NMS,1QX3,2J30,2J31,2J32,2J33,3H0E,3ITN,3PCX,3PD0,3PD1,4EHA,4EHD,4EHF,4EHH,4EHK,4EHL,4EHN,4JJE,4JQY,4JQZ,4JR0,4PRY,4PS0,4QTX,4QTY,4QU0,4QU5,4QU9,4QUA,4QUB,4QUD,4QUJ,4QUL,5I9B,5IAJ,5IAK,5IAS,5IBC,5IBP,3DEH,3DEI,3DEJ,3DEK,4QU8,4QUE,4QUG,4QUH,4QUI,5I9T,5IAB,5IAE,5IAG,5IAN,5IAR,5IBR,1I3O,7XN4,7XN5,7XN6	XIAP,PARP1,BIRC2,CASP9,CDKN1A,CRYAB,MDM2,BIRC5,BIRC6,CASP6,CASP8,CFLAR,HCLS1,MAPT,AFP,ARNT,BID,BIRC3,BIRC7,CASP2,CTTN	Apoptosis, PANoptosis, Pyroptosis, Autophagy, Stem cell and neural differentiation	Cancer (Colorectal, Lung, Prostate, Breast, Hepatocellular, Pancreatic, Ovarian, Gastric, Esophageal, Endometrial, Brain), Type 1 and 2 diabetes mellitus, Systemic lupus erythematosus, Familial Alzheimer disease, Amyotrophic lateral sclerosis, Hypertension, Acute myeloid leukemia, Crohn’s disease, Age-related macular degeneration, Osteoporosis, Parkinson disease (late onset), Ataxia-telangiectasia, Complementation group Fanconi anemia, Ischemic stroke, Rheumatoid arthritis, Familial meningioma, Duchenne muscular dystrophy, Multiple sclerosis, Retinitis pigmentosa, Nonpapillary renal cell carcinoma, Malaria, Cystic fibrosis, Gastrointestinal stromal tumor, Neural tube defects, Cone-Rod dystrophy, Leptin deficiency, Interstitial lung disease, Medulloblastoma and retinoblastoma, Dilated cardiomyopathy, Schizophrenia, Nephrological diseases, Head and neck squamous cell carcinoma, Lung cancer susceptibility, Autism spectrum disorder, Chronic lymphocytic leukemia, Multiple myeloma, Ulcerative colitis, Tuberous sclerosis, Asthma, Tatton-Brown-Rahman syndrome, Familial adenomatous polyposis	E25K^lb^, E34K^lb^, R75H^lb^, R101H^lb^, R110H^lb^	Glycyrrhizic acid, Minocycline, Oleandrin, Pamidronic acid, Emricasan, Z-VAD, VX-166, Calpain	^9,11,72,100^
CASP4	6NRY,7WR0,7WR6,7WR1,6KMZ,8SPB,8J6K,7WR4,7WR5	CASP5	Apoptosis, Pyroptosis, ER stress	Neuroblastoma, Cancer (Colorectal, Lung, Prostate, Breast, Hepatocellular, Pancreatic, Ovarian, Gastric, Esophageal, Brain), Familial Alzheimer disease, Hypertension, Age-related macular degeneration, Parkinson disease (late onset), Complementation group Fanconi anemia, Nonpapillary renal cell carcinoma, Gastrointestinal stromal tumor, Neural tube defects, Cone-Rod dystrophy, Schizophrenia, Lung cancer susceptibility, Multiple myeloma	E228D, E284D, D47N	Incadronic acid, Emricasan, Z-VAD, VX-166	^11,20^
CASP5	–	ARHGAP44, CASP4,LACRT	Pyroptosis	Cancer (Colorectal, Lung, Prostate, Breast, Pancreatic, Ovarian, Gastric, Brain, Endometrial), Nonpapillary renal cell carcinoma, Lynch syndrome, Retinoblastoma, Familial Mediterranean fever, Ulcerative colitis	R11H^lb^, R24H^lb^, E164Q^lb^, E240Q^lb^, E324Q^lb^, E382Q^lb^, E395Q^lb^		^11,20^
CASP6	3P4U,3P45,3QNW,8EG5,8EG6,3NKF,3V6L,4IYR,2WDP,3K7E,3NR2,3OD5,3S70,4EJF,4FXO,4HVA,4N5D,4N6G,4N7J,4N7M,4NBK,4NBL,4NBN,6DEU,6DEV,8F78,8F96,8F98,8F99,8F9A,8F9B,8F9C,8F9D,8FBV,3S8E,3V6M,8F97	CASP3,CASP8,MAPT,STUB1,USP15	Apoptosis, PANoptosis, Synaptic loss and disfunction, B-cell proliferation	Cancer (Colorectal, Lung, Prostate, Breast, Hepatocellular, Pancreatic, Ovarian, Gastric, Endometrial, Esophageal, Brain), Familial Alzheimer disease, Amyotrophic lateral sclerosis, Interstitial lung disease, Lung cancer susceptibility, Head and neck squamous cell carcinoma, Multiple myeloma, Chronic lymphocytic leukemia	-	Emricasan, Z-VAD, VX-166	^11,26,272^
CASP7	2QLF,3IBC,3IBF,5IC6,6X8J,6X8L,2QL5,2QL7,2QL9,2QLB,2QLJ,3EDR,4JB8,4LSZ,4ZVO,4ZVP,4ZVQ,4ZVR,4ZVS,4ZVT,4ZVU,5K20,6CL1,6CL2,1K86,1K88,1SHJ,1SHL,4FDL,4FEA,4HQ0,4HQR,4JJ8,4JR1,4JR2,5V6U,5V6Z,8DGZ,8DJ3,1F1J,1GQF,3H1P,3R5K,1I51,1I4O,1KMC,7WZS	ATXN3,BIRC2,BIRC3,BIRC5, BIRC6,CASP8,CCT2,CRYAB, FBXW11,HNRNPH1,HSPA5,NFE2L2, PARP1,PTGES3, RB1,SAT1,TRIM25,XIAP	Apoptosis, PANoptosis, Bone development, Dentinogenesis	Cancer (Colorectal, Lung, Prostate, Breast, Hepatocellular, Pancreatic, Ovarian, Gastric, Esophageal, Endometrial), Familial Alzheimer disease, Crohn’s disease, Age-related macular degeneration, Parkinson disease (late onset), Type 1 diabetes mellitus, Rheumatoid arthritis, Ischemic stroke, Duchenne muscular dystrophy, Nonpapillary renal cell carcinoma, Retinitis pigmentosa, Osteoarthritis, Chronic nephrological diseases, Chronic lymphocytic leukemia, Head and Neck squamous cell carcinoma, Tatton-Brown-Rahman syndrome, Lung cancer susceptibility	D230E, T244S, D255E, D263E, D288E, D340E	Fica, Emricasan, Incadronic acid, Z-VAD, VX-166	^9^^,11,273^
CASP8	1F9E,1QDU,1QTN,2C2Z,3KJN,3KJQ,6X8H,2K7Z,4JJ7,4PRZ,4ZBW,5H31,5H33,6AGW,7LVJ,7LVM,2Y1L,4PS1,5L08,6PX9,1I4E,5JQE,2FUN,3H11,7DEE,8YM4,8YM5,8YM6,8YNK,8YNL,8YNM,8YNN,8YBX,8YD7,8YD8,8YNI	BCAP31,BCL2,BID,BIRC2,BIRC3,CASP2, CASP3,CASP6,CASP7,CASP9,CASP10,CFLAR,CUL3,DEDD,DEDD2,FADD,FAF1, FAS,FASLG,GMEB1,HECTD3,HIP1,IKBKB, IL1B,MAP1LC3B,NLRC4,NOL3,PEA15, PIK3C3,PML,PRDX6,PTEN,PYCARD, RIPK1,RIPK3,RNF34,SQSTM1,SRC, TICAM1,TNF,TNFAIP3,TNFRSF10A, TNFRSF10B,TNFSF10,TRADD, TRAF1,TRAF2,TRAF6,XIAP	Apoptosis, Pyroptosis, PANoptosis, Necroptosis, Lens cell differentiation, Terminal keratinocytes differentiation, Erythrocytes & platelet formation, T & B cell proliferation	Cancer (Colorectal, Lung, Prostate, Breast, Hepatocellular, Pancreatic, Gastric, Ovarian, Esophageal, Brain, Endometrial), Type 2 diabetes mellitus, Systemic lupus erythematosus, Amyotrophic lateral sclerosis, Familial Alzheimer disease, Acute myeloid leukemia, Crohn’s disease, Age-related macular degeneration, Osteoporosis, Parkinson disease (late onset), Ataxia-telangiectasia, Type 1 diabetes mellitus, Multiple sclerosis, Familial meningioma, Complementation group Fanconi anemia, Rheumatoid arthritis, Ischemic stroke, Nonpapillary renal cell carcinoma, Cystic fibrosis, Type I neurofibromatosis, Pulmonary hypertension, Lynch syndrome, Neural tube defects, Proteasome-associated autoinflammatory syndrome, Cone-Rod dystrophy, Leptin deficiency, Osteoarthritis, Interstitial lung disease, Medulloblastoma, Retinoblastoma, Autoimmune lymphoproliferative syndrome, Asthma, Head and neck squamous cell carcinoma, Lung cancer susceptibility, Chronic lymphocytic leukemia, Multiple myeloma, Ulcerative colitis, Autism spectrum disorder, Tuberous sclerosis, Familial Mediterranean fever, Tatton-Brown-Rahman syndrome, Familial adenomatous polyposis	K14R, D71H, D80H, D86H, D182H, D201H, D216H, D260H, D262H, D270H, D285H, D296H, D302H, D329H, D344H	Bardoxolone, Bryostatin 1, AN-9, Trichostatin A, Oleandrin, Emricasan, Z-VAD, VX-166	^11,36,69–71,102,105,106,148^
CASP9	2AR9,1JXQ,3V3K,3YGS,4RHW,5WVC,1NW9,3D9T,5JUY,5WVE	ABL1,APAF1,ARNT,BCL2L1,BIRC2,BIRC3, BIRC5,BIRC6,BIRC7,BIRC8,CASP3,CASP8, HECTD3,NLRP1,XIAP	Apoptosis, Synaptic loss, Erythropoiesis, Embryonic development & leukemia, Muscle differentiation	Cancer (Colorectal, Lung, Prostate, Breast, Hepatocellular, Pancreatic, Ovarian, Gastric, Esophageal, Endometrial, Brain), Familial Alzheimer disease, Amyotrophic lateral sclerosis, Acute myeloid leukemia, Crohn’s disease, Osteoporosis, Parkinson disease (late onset), Type 1 diabetes mellitus, Ataxia-telangiectasia, Ischemic stroke, Nonpapillary renal cell carcinoma, Neural tube defects, Cone-Rod dystrophy, Osteoarthritis, Medulloblastoma, Schizophrenia, Dilated cardiomyopathy, Head and neck squamous cell carcinoma, Lung cancer susceptibility, Multiple myeloma, Autism spectrum disorder, Chronic lymphocytic leukemia, Ulcerative colitis, Tuberous sclerosis, Tatton-Brown-Rahman syndrome	L23V, A28V, L106V, T216N, T283N, T366N	Pamidronic acid, Emricasan, Z-VAD, VX-166	^11,41,42^
CASP10	–	CASP8,CFLAR,FADD,FAS,PRDX6,RIOK3, RNF34,TNFRSF10B,TNFRSF1A,XIAP	-	Cancer (Gastric, Colorectal, Lung, Prostate, Breast, Pancreatic, Ovarian, Brain, Esophageal), Systemic lupus erythematosus, Amyotrophic lateral sclerosis, Crohn’s disease, Rheumatoid arthritis, Pfeiffer syndrome, Lynch syndrome, Immune deficiency disease, Dilated cardiomyopathy, Retinoblastoma, Medulloblastoma, Autoimmune lymphoproliferative syndrome, Chronic lymphocytic leukemia, Asthma, Multiple myeloma, Familial adenomatous polyposis, Ulcerative colitis	K17E, V343I, A347V, V367I, A371V, Y379C, Y403C, V410I, A414V, Y446C, L455I, L479I, L522I	Emricasan, Z-VAD, VX-166	^11,274^
CASP12	–	MAGEA3	ER stress	Cancer (Colorectal, Breast, Ovarian, Gastric), Type 2 diabetes mellitus, Malaria, Cardiovascular disease, Tuberous sclerosis	S53N^lb^	Emricasan, Z-VAD, VX-166	^275,276^
CASP14	–	ALDH3A1,CCNF,FCF1,FTH1,MEOX2, MYC,TBC1D22B,TP53,ZIC1,ZRANB1	Keratinocyte differentiation	Cancer (Prostate, Breast, Pancreatic, Gastric, Brain), Ischemic stroke, Head and neck squamous cell carcinoma, Autism spectrum disorder	L65V R179Q^lb^	Emricasan, Z-VAD, VX-166	^277,278,279^

^a^Listed PDB entries as of 11/01/2024.

^b^Retrieved from BioGRID v4.4 database (accessed 03/28/2024). Proteins that support at least two experimental evidences were included.

^c^Retrieved from MalaCards database (accessed 03/28/2024). Diseases that obtained a minimum of 75 MIFTS score were only included.

^d^Retrieved from ClinVar database (accessed 03/28/2024). The superscript “lb” refers to likely benign. Mutations that did not carry the “lb” superscript are either pathogenic, benign, or disease-associating mutation.

^e^Retrieved from DrugBank database (accessed 03/28/2024).

Caspases belong to the cysteinyl protease family, which utilizes a histidine-cysteine catalytic dyad to hydrolyze peptide bonds. These proteases cleave aspartic acid residues with stringent specificity at the P1 site, which has led to their designation as cysteine-dependent aspartate-specific proteases (caspases)^30–32^^,^. Caspases are expressed in both immune and non-immune cells, and most are constitutively expressed during homeostasis^33,34^. During apoptosis, caspase activation occurs through extrinsic and intrinsic pathways, where the initiator caspase-8 is activated through external factors (e.g., TRAIL and FAS ligands^35–40^), or caspase-9 is activated through internal factors (e.g., DNA damage leading to cytochrome C release and apoptotic protease-activating factor-1 (APAF-1) oligomerization^41–44^). Upon activation, caspase-8 or -9 cleaves and activates the executioner caspase-3 and -7 to drive cell death^37–40,43,45,46^. In contrast, the lytic necroptosis pathway is activated when caspase-8 is absent or has its enzymatic activity restricted due to mutation or regulation^47–55^. The inhibition of caspase-8 prevents apoptosis and allows the activation of necroptosis by enabling receptor-interacting serine/threonine protein kinases (RIPKs) to drive the phosphorylation and activation of the pore-forming molecule mixed lineage kinase domain-like pseudokinase (MLKL), leading to membrane disruption and cell death^47,48,50,52,56–61^. Furthermore, the lytic innate immune pyroptosis pathway can be triggered through canonical (caspase-1-mediated) and non-canonical (caspase-4/5/11-mediated) pathways^14,62–64^. In the canonical pathway, innate immune sensors, such as nucleotide-binding domain, leucine rich-containing family, pyrin domain-containing (NLRP) 1 or NLRP3, are activated by PAMPs or DAMPs, triggering the formation of a multiprotein complex called the inflammasome^14–17^. The inflammasome recruits the adaptor molecule apoptosis-associated speck-like protein containing a caspase activation and recruitment domain (ASC) and pro-caspase-1. Inflammasome formation facilitates auto-proteolytic cleavage of caspase-1, which subsequently cleaves downstream molecules, such as the pro-inflammatory cytokines IL-1β and IL-18 and the pore-forming molecule gasdermin D (GSDMD). In the non-canonical pathway, caspase-11 in mice, or caspase-4 or -5 in humans, directly cleaves GSDMD to trigger activation of NLRP3 inflammasome-dependent caspase-1 maturation and cytokine release^18–20,62,64–66^.

Data from the past decades have identified extensive crosstalk and conserved functions among the caspases^27,67–73^. These findings identified a major gap in our understanding of the molecular mechanisms of cell death and led to the conceptualization of PANoptosis, a lytic, innate immune cell death pathway initiated by innate immune sensors and driven by caspases and RIPKs through molecular complexes known as PANoptosomes. PANoptosis is characterized by the activation of several caspases, including caspase-1, -3, -7, and -8^21–27,74^, though others may also be involved, and microscopy and co-immunoprecipitation studies have provided extensive evidence that multiple caspases, including caspase-1 and -8, are key components of PANoptosomes^21–26,75^. PANoptosis has also been associated with a range of infectious and inflammatory diseases and cancers^21–27,70,71,73,74,76–83^.

Collectively, caspases have central functions in multiple cell death pathways, making them potential therapeutic targets for a range of conditions, including infectious diseases, neurodegeneration, inflammation, metabolic disease, and cancer^34,84–86^. Therefore, it is critical to understand the molecular functions of caspases. In this review, we discuss the central roles of caspases in innate immunity with a focus on their 3D structures and their substrates to illustrate the interaction between active sites and exosite regions of the respective substrates (Table 1). We also discuss the limited biological validations performed to date for various tetrapeptides that define substrate specificity, which often limits our ability to understand their physiological functions. Furthermore, we highlight how the integral roles of caspases in both non-lytic and innate immune lytic cell death and inflammation have made them attractive therapeutic targets. Recent advances in biochemical and structural studies of caspases have provided a deeper understanding of their functions, improving our ability to translate this knowledge toward the development of novel therapeutics.

## Caspase classification and domain organization

**Fig. 1:**
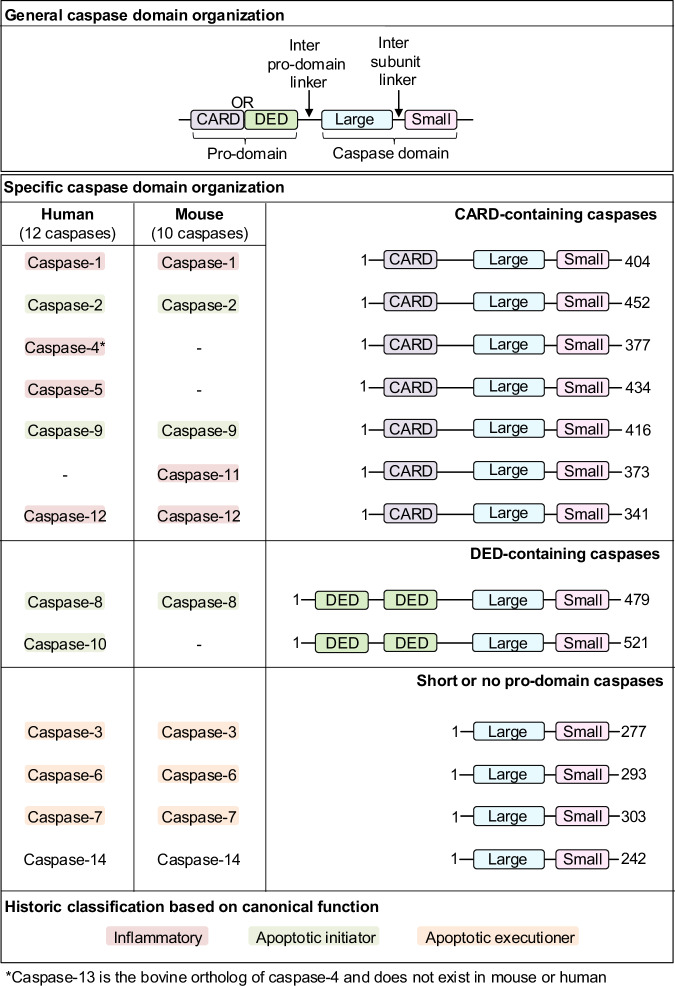
Domain organization of caspases. General domain organization, followed by human and mouse caspase classification based on domain organization. Domain boundaries were taken from human caspase UniprotKB annotations. The historic classifications based on canonical function are shown by the highlighting color on the caspase name. CARD caspase activation and recruitment domain, DED death effector domain.

Caspases are expressed across all kingdoms of life. However, the number of caspases varies across species^29,87–89^. For example, humans have 12 caspases; mice carry 10 (Fig. 1)^87,90,91^. These variations in the number of caspases across species highlight the evolutionary pressure that has shaped caspase repertoires in various lineages. Caspases are historically categorized into distinct sub-families based on gene duplication, structure, substrate specificity, and functionality. Based on the substrate sequence specificity, caspases can be divided into group I (caspase-1, -4, -14: preference of (W/L/Y)EHD), group II (caspase-2, -3, -7: preference of DEXD), and group III (caspase-6, -8, -9, -10: preference of (L/V/I)EXD), where “X” refers to a variable position that may be fulfilled by multiple amino acids^92^. This method of caspase substrate-based classification is discussed further in the section “Caspase substrate recognition”.

Caspase-mediated activation cascades can promote lytic or non-lytic cell death and cellular homeostasis, where cellular fate depends on the molecular characteristics of the caspases involved, including their substrate specificity and the availability of downstream partner molecules^93^. Additionally, supramolecular complexes induced by innate immune activation, such as inflammasomes or PANoptosomes, activate various caspases^6,14,21^. Given these functions, caspases were historically classified into functional groups, including the inflammatory caspases (caspase-1, -4, -5, and -11) and the apoptotic caspases. Apoptotic caspases were further subdivided into apoptotic initiators (caspase-2, -8, -9, and -10) and apoptotic executioners (caspase-3, -6, and -7)^34,87^. However, recent studies have shown extensive connections between the roles of caspases, which have been reviewed elsewhere^8,94–96^. In addition, groups have suggested that caspases do not neatly fit into apoptotic vs inflammatory categories, and that caspases can have multi-faceted roles^97^. Studies from the past decades suggest that historic functional classifications may not fully represent the complexity of the roles of caspases in biological processes. For example, apoptotic caspases are now known to also regulate inflammatory cell death. Caspase-3, an apoptotic executioner, cleaves GSDME in the linker region at the DMPD recognition site to release an N-terminal fragment, which triggers inflammatory, lytic cell death in a similar mechanism to that of the GSDMD N-terminus^72,98,99^. Caspase-3 also cleaves GSDMD; however, this cleavage occurs within the N-terminal domain at the DAMD recognition site rather than at the linker region, leading to functional inhibition of GSDMD^100,101^. Furthermore, caspase-8 acts as an upstream regulator of the NLRP3 inflammasome to control inflammatory cell death^69^. Additionally, caspase-8 can cleave GSDMD to promote the formation of pores in the membrane^102–105^, and caspase-8 can cleave IL-1β at the same site as caspase-1 to produce a mature form of the cytokine^106^. The metabolite α-ketoglutarate also induces lytic cell death through caspase-8–mediated cleavage of GSDMC^107^. Furthermore, the C362A mutation in caspase-8 renders it inactive, altering cell death dynamics in MLKL-deficient cells, where necroptosis is impaired. In these cells, GSDMD is cleaved by caspase-1, as expected, but caspase-3 and -7 can also contribute to its cleavage^61,105,108,109^, highlighting additional complex interplay between the caspases in regulating cell death. In the case of PANoptosis, there is activation of caspase-1, -8, -3, and -7^21–27,74^, and a regulatory role for caspase-6^26^, implying additional interconnections among these caspases. Adding further complication to the historic functional classification scheme, the potential roles of caspase-2, -9, and -10 in inflammation remain poorly understood^110–112^. Additionally, caspase-12 is considered an inflammatory caspase^113,114^, and it is polymorphic in nature due to a stop codon, resulting in the production of both active and inactive forms^115–117^. In humans, it is generally considered inactive. Caspase-14 is associated with epidermal differentiation and skin barrier formation, rather than with traditional caspase functions^118–121^. Despite these unique functions, caspase-14 is often classified as an apoptotic executioner caspase due to its putative short pro-domain. However, the role of caspase-14 during apoptosis appears to be minimal, as it is not known to cleave classical caspase substrates^122^. Hence, it is not strictly classified as inflammatory or apoptotic^123,124^, and its role in inflammation is yet to be established.

Collectively, this growing evidence from recent decades suggests that the classic functional categorization of caspases can be enriched by incorporating a structural perspective that addresses their multi-dimensional roles across cellular contexts. Based on this understanding, multiple caspase categorization strategies have now been introduced, including those based on function, substrate specificity, and the length of their pro-domains, distinguishing between long and short pro-domain-containing caspases^92^. In the current review, we further emphasize the utility of a classification system for caspases based on their domain architecture, categorizing them into three broad groups: caspase activation and recruitment domain (CARD)-containing caspases (caspase-1, -2, -4, -5, -9, -11, -12), death effector domain (DED)-containing caspases (caspase-8 and -10), and the short/no pro-domain-containing caspases (caspase-3, -6, -7, -14) (Fig. 1). This approach aims to integrate both structural and functional insights into the roles of caspases in cell death and other cellular processes.

## Caspase activation in innate immunity and cell death

Across caspase sub-families, their critical roles in innate immunity have been identified through their involvement in cell death. There is growing evidence that different types of cell death play important roles in innate immunity, including regulating inflammatory response, modulating cytokine production and release, driving anti-viral defense, enabling specialized immune cells to kill microbes, and promoting immune tolerance^125–129^. Apoptosis is the most well-studied non-lytic cell death pathway, and it is critical for organismal development, tissue homeostasis, and clearance of dead or infected cells through phagocytosis^12,130^. It is a mechanism of ‘silent’ cell death, where caspases hydrolyze cellular components, resulting in DNA degradation, nuclear shrinkage, membrane blebbing, and release of apoptotic bodies^12,13,131,132^. Different signals can initiate apoptosis via either intrinsic or extrinsic pathways, which have been extensively reviewed previously^11,133,134^. These pathways depend on caspase activity and the subsequent cleavage of specific substrates^135,136^. The intrinsic pathway of apoptosis is triggered when mitochondria are compromised in response to endogenous or exogenous stressors, such as intracellular pathogens. In intrinsic apoptosis, BCL-2 antagonist or killer (BAK) and/or BCL-2–associated X (BAX) promotes mitochondrial outer membrane permeabilization (MOMP), causing the loss of mitochondrial integrity^137^. This leads to the release of the intermembrane space protein, cytochrome c, promoting the assembly of a multiprotein complex that includes APAF-1 and cytochrome c. This complex is called the apoptosome, and it was one of the earliest cell death-inducing complexes discovered. Following release from the mitochondria, cytochrome c interacts with APAF-1 in an ATP-dependent manner to form a wheel-shaped, heptameric ring structure^11,138,139^. In this case, APAF-1 forms a scaffold to recruit caspase-9 monomers, which are then dimerized to form the catalytically active enzyme^140–142^. When activated, caspase-9 cleaves and activates caspase-3 and caspase-7, initiating the apoptotic cascade and cell death^11,138,140–142^. While caspase-3 and caspase-7 also act as executioners in the extrinsic apoptosis pathway, the upstream events that lead to their activation differ from those in the intrinsic pathway. Extrinsic apoptosis is driven by extracellular ligand-induced death receptor (DR) signaling. This is mediated by a group of transmembrane DRs, which include TNF receptor 1 (TNFR1/CD120a), APO-1 (commonly called Fas or CD95), DR3, DR4, and DR5^90,143–145^. The binding of these receptors to their cognate trimeric ligands induces apoptosis via the extrinsic pathway, which is characterized by receptor oligomerization and recruitment of the downstream adaptors, FAS-associated death domain (FADD) or TNFR1-associated death domain (TRADD). These adaptors bridge the initiator caspases, such as caspase-8 or -10, to form a macromolecular death-inducing signaling complex (DISC)^35,146–148^; caspase-8 then activates executioner caspases, inducing cell death. In addition, activation of caspases via the granzyme B pathway is a key mechanism of cell death during cytotoxic T lymphocyte (CTL)/natural killer cell (NK)-mediated cytotoxicity^132,149^. Dysregulation of apoptosis has been linked to several pathological conditions^11^.

Beyond apoptosis, caspases also drive innate immune, lytic, inflammatory cell death, including pyroptosis and PANoptosis. Pyroptosis is a caspase-1-mediated lytic form of cell death, which is distinct from the non-lytic apoptotic pathway. It was characterized as an inflammatory form of cell death because caspase-1 induces the maturation and release of the proinflammatory cytokine IL-1β^10,150^. Caspase-1 activation is regulated downstream of innate immune pattern recognition receptors (PRRs) sensing their cognate ligands to form the inflammasome, a molecular platform that drives caspase-1 activation^3,14^. Multiple PRRs, including NLRP3, NLRP1, NLR family CARD-containing protein 4 (NLRC4), absent in melanoma 2 (AIM2), Pyrin, and others, can induce inflammasome formation and caspase-1 activation^3^. NLRP3 is the most well-studied inflammasome sensor, and it is activated by several innate immune stimuli, such as lipopolysaccharide (LPS, a gram-negative bacterial outer membrane component), ATP, nigericin (an antibiotic derived from *Streptomyces hygroscopicus*), aluminum salts, monosodium urate crystals, and other triggers^15–17,151,152^.

In addition to caspase-1, it is now known that other inflammatory caspases, including caspase-11 in mice and caspase-4/-5 in humans, are also involved in inflammasome formation in response to intracellular LPS^63,153–156^. Discerning the roles of caspase-1 and -11 in host defense against bacterial infections was initially complicated by early efforts to generate a caspase-1-knockout mouse, as the genetic targeting of caspase-1 in these mice also disrupted the function of the closely linked caspase-11 gene. These doubly deficient mice were resistant to endotoxic shock and lethality induced by LPS^157,158^. However, when the genetic deletion of caspase-11 alone was achieved, it was found that these mice were also resistant to LPS-induced septic shock^62,159^, suggesting that the LPS response in the original caspase-1/-11 doubly deficient mice was due to caspase-11. It is now known that caspase-11 triggers lytic cell death in macrophages and dendritic cells during gram-negative bacterial infections^62,160,161^. Unlike caspase-1, which requires the assembly of the inflammasome complex for activation, caspase-11 can be directly activated by LPS to undergo self-oligomerization and autoactivation^63,153,155^. Caspases process and multimerize pore-forming gasdermin family members, such as GSDMD, to drive lytic cell death^18,20,66,72,100,102,103,162,163^. Cleavage of the linker region between the N- and C-terminal domains of GSDMD liberates the active N-terminus from the autoinhibiting C-terminal domain^18,20^, allowing the active N-terminal GSDMD fragments to translocate to the plasma membrane and form oligomeric pores, which in turn, leads to the extracellular release of inflammatory cytokines and DAMPs^66,162,163^.

In addition to their roles in apoptosis and pyroptosis, caspases are also key regulators of PANoptosis^6,11,96,164^. Bacterial and viral pathogens such as influenza A virus (IAV), herpes simplex virus type-1 (HSV1), or *Francisella novicida*, as well as exposure to other PAMPs, DAMPs, cytokines, and homeostatic changes, induce the activation of PANoptotic molecules, including caspases (e.g., caspase-1, -8, -3, and -7) and RIPKs^21–27,73,74,76–83^. The prototypical example of PANoptosis activation is seen during IAV infection. In this case, ZBP1 acts as the innate immune sensor for IAV infection and forms the ZBP1-PANoptosome, containing NLRP3, ASC, RIPK3, RIPK1, caspase-8, and caspase-6, to activate the NLRP3 inflammasome, caspase-1, -8, -3, and -7, RIPK3, and downstream executioners to drive inflammatory cell death^26,27,75^. Deletion of individual downstream components, such as caspase-1 for pyroptosis and MLKL for necroptosis, does not protect against IAV-induced cell death in macrophages^27,76^. This observation differentiates PANoptosis from other forms of lytic cell death. Indeed, deletion of the upstream sensor ZBP1 completely protects macrophages from IAV-induced cell death and caspase activation^27^. The ZBP1-PANoptosome has also been proposed as a therapeutic target for cancer treatment, as it can be induced by treatment with IFN and a nuclear export inhibitor to drive tumor regression^77^. Several other innate immune sensors have been implicated in PANoptosome formation in response to bacteria, viruses, fungi, and sterile stimuli, including AIM2, NLRC5, NLRP12, NLRP3, and RIPK1, and caspases have been identified as key components of each of the PANoptosomes characterized to date^21–25^^,165^. The collective literature highlights PANoptosis as a distinct pathway with clearly defined genetic and biochemical features.

Together, these studies have defined the central role of a diverse array of caspases in driving cell death as part of the innate immune response against infections, inflammatory conditions, and cancers.

## Features of the caspase catalytic domain

The primary function of caspases is to cleave their substrates. During the activation of cell death, development, and innate immune pathways, caspases cleave several hundred substrates to dismantle the cell^136^. To carry out these functions, most caspases contain a proteolytic C-terminal catalytic domain with large (17–20 kDa) and small (10–12 kDa) subunits connected by an inter-subunit linker (Fig. 1)^136^. The catalytic domain must undergo structural changes and forms a substrate-binding pocket that enables enzymatic activity. In addition to a catalytic domain, some caspases also contain a non-enzymatic pro-domain (i.e., CARD or DED) in the N-terminal region, which facilitates protein–protein interactions^166–168^.

**Fig. 2:**
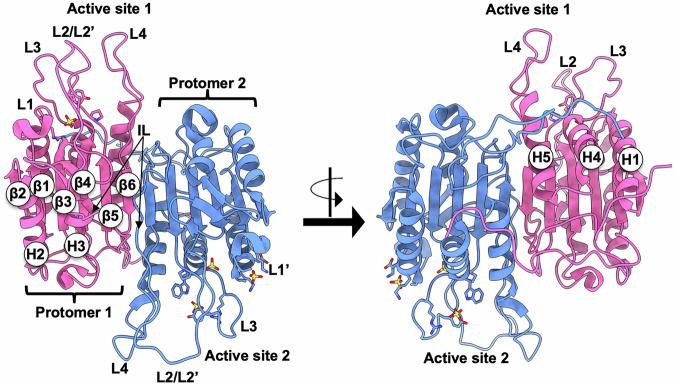
Structural representation of caspase dimer. Caspase zymogen (PDB ID: 1GQF) with each protomer unit of the caspase heterodimer colored in pink and cornflower blue, respectively. The active site loops L1–L4, intersubunit linker (L2/L2’ and IL), and L1’ from the second heterodimer are shown. Positions of β strands β1–β6 and helices H1–H5 are indicated.

Different caspases undergo activation in diverse ways, many of which are driven by the structural aspects of the proteins. Executioner caspases exist as homodimers in the inactive state, and they are activated upon cleavage of the large and small subunits in the C-terminus. In contrast, inactive initiator caspases exist as monomers and become activated upon dimerization^169^. Most effector pro-caspases undergo proteolysis to become active; the active caspases in their homodimer/heterodimer configurations form a two-fold axis that is associated with the pair of catalytic domains and forms two active sites^170^. Atomic structures show that the matured caspase protomer is formed by the folding of large and small subunits into a single domain in a head-to-tail manner; one protomer provides an interface that stabilizes the active conformation of the adjacent protomer, which is referred to as the caspase-hemoglobinase fold^92,171^ (Fig. 2). The fold of the caspase protomer is composed of six β-sheets (β1–β6) with five α-helices (H1–H5) on the protein surface. The helices H2 and H3 reside on the same face of the protein, whereas helices H1, H4, and H5 are on the opposite side (Fig. 2). The protomer also contains three short β-sheets near the surface of the active site that undergo significant structural changes upon activation, while no changes occur in the core 6 β-strands^92^.

Structural studies using peptide-bound caspases have found that they share a similarly shaped active site. The binding site contains five loops that connect α-helices and β-sheets in the vicinity of the active site, referred to as L1–L4 and L2’. The L1 loop constitutes one side of the groove by connecting β1 and H1, while the L2 loop, which harbors the catalytic residue Cys, and L2’ form the intersubunit linker (IL) connecting β4 in the large subunit to β5 in the small subunit. Additionally, the L3 loop connects β5 and H4, and the L4 loop represents the other side of the active site by connecting H5 and β6^172,173^ (Fig. 2). During caspase maturation, the IL is cleaved, resulting in the L2’ loop of one protomer interacting with the L2 and L4 loops of the second protomer and stabilizing the active conformation^173^. Hence, a single protomer cannot be active, because it lacks the cross-protomer interactions in the active site loops.

**Fig. 3:**
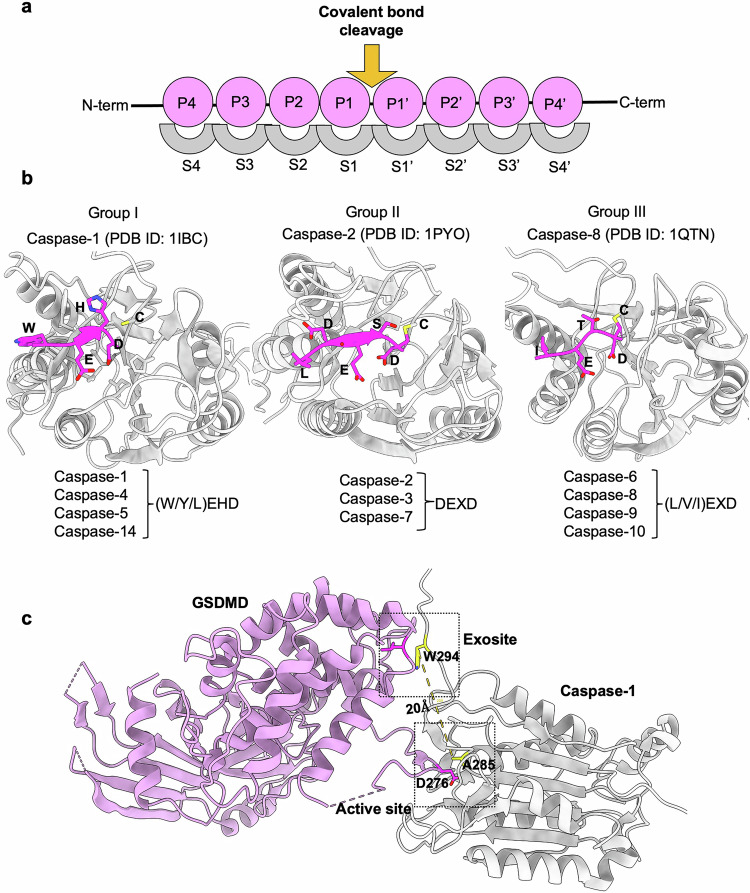
Substrate binding to the caspase active site and exosite. **a** The naming convention for substrate residues interacting with the caspase active site^175^. The substrate residues to the N-terminal side of the cleaved peptide bond are named P1–P4, and the ones to the C-terminal side are named P1’–P4’. Several substrate residues interact with proteases with specific interacting pockets, which are named S (subsite) and numbered following the same logic used as the P1–P4 and P1’–P4’ in the substrate. Adapted from^90^. **b** Substrate binding to the caspase active site. Illustration of three classes of caspase substrate-binding specificities on the protein active site (S4–S1) to the substrate (P4–P1) positions; the caspase structures are shown in gray, and the substrates are shown in magenta. Caspase-1 (PDB ID: 1IBC), caspase-2 (PDB ID: 1PYO), and caspase-8 (PDB ID: 1QTN) are provided as examples. **c** Illustration of the human caspase-1:murine GSDMD complex structure (PDB ID: 6VIE) showing the active site and an exosite 20 Å away from the active site region.

Among all the loops, L1 and L3 display relatively conserved lengths and compositions across all mammalian caspases, whereas L2 and L4 are highly divergent^174^. These five loops determine the sequence specificity of the caspase substrates. Within the active site, the specificity-determining pocket accepts specific residues, named the P4–P1 sites, on the substrate; the convention for naming substrate residues interacting with any protease active site is based on Schechter and Berger^175^. Generally, S1–S4, the corresponding subsites (S) on the caspase, are the key sites that determine the specificity of the substrate recognition. The primary specificity site (S1) is the deep and basic pocket in the caspase that accommodates the aspartic acid residue from the substrate, leading to the cleavage site in the substrate (referred to as P1) (Fig. 3a)^176^.

## Caspase substrate recognition

The available protein structures of caspases in the presence of peptide-based inhibitors have shown that most caspases share a similar conformation in their substrate-binding groove and interact in similar manners. Through Nov 2024, 329 experimental 3D structures of human caspases had been deposited in protein data bank (PDB) (Table 1), along with hundreds of caspase substrates. Most peptide-bound caspase active site structures show similar structural organization, leading to a hypothesis that the substrate-binding pocket of caspases is pre-formed^170^. However, the crystal structure of apo caspase-7 (an unbound form of the enzyme) shows that the loops forming the active site are significantly different compared to its holo form (peptide-bound form of the enzyme), suggesting that a conformational change occurs upon ligand binding, enabling the enzyme to effectively perform its catalytic function^177^.

In general, the caspase active site forms a deep and basic pocket to accommodate the aspartic acid residue of the substrate (P1), leading to substrate cleavage (Fig. 3a). Caspases can be distinguished by their ideal binding pocket P4-P3-P2-P1 residues, which correspond to the S4-S3-S2-S1 subsites. The S1 and S3 sites are nearly identical among all caspases, and the identities of the S2 and S4 sites are highly conserved^174^. Numerous structures of peptide-bound caspases have identified that all caspases require an aspartate residue at the P1 position to recognize their tetrapeptides. The S1 subsite renders a cavity on the surface of the binding groove that accommodates its substrate’s aspartate residue; hence, the prerequisite for having an aspartate residue at the P1 position is highly conserved in caspases^90,136,174^. However, the enzyme specificity of the substrate is predominantly determined by the amino acid preference at its P4 position. Most structural studies have focused on identifying novel substrates based on the P1–P4 site in substrate-bound caspase structures, which has led to the development of substrates that only target the P1–P4 sites in various caspases^92,136^.

A combination of global strategies, such as the use of substrates equipped with reporter groups, potential scanning-substrate combinatorial libraries, microarray techniques, phage display libraries, and proteomic-based techniques, have enabled the identification of several peptide-based substrate targets for individual caspases^31,178–182^. However, the number of substrates identified for each of the caspases varies significantly. For example, only a few dozen targets have been reported for caspases-4, -5, -9, and -14, while hundreds of targets for caspases-1, -2, -3, -6, -7, and -8 have been found^181^. Additionally, proteome degradation studies characterizing the kinetics of target cleavage show that each caspase has a preferred substrate group, which can sometimes overlap with the preferred substrate group of other caspases^181^. However, the rate of cleavage for these caspases varies over 500-fold within each group. Synthetic tetrapeptides have been suggested for use in therapeutics, based on their ability to interact with caspases. Detailed literature on caspases and their tetrapeptide substrates has been reviewed and characterized elsewhere^31,181–183^, but these peptide-based substrates have limitations, as they lack selectivity, and several potential substrates require functional validation. Together, these issues lead to uncertainties about the therapeutic effects of tetrapeptides.

Based on their ability to recognize specific tetrapeptide substrate sequences, caspases are classified into three groups: Group I (caspase-1, -4, -5, -14), Group II (caspase-2, -3, -7), and Group III (caspase-6, -8, -9, -10) (Fig. 3b)^92^. Group I caspases recognize a specific amino acid sequence called the (W/Y/L)EHD sequence^92^. In this sequence, P4 may be W, Y, or L, whereas P3, P2, and P1 must be E, H, and D, respectively. Group II caspases recognize the DEXD sequence, where P2 may be any amino acid (X)^92^. Caspases in Group III recognize the (L/V/I)EXD sequence, where P4 may be either L, V, or I and P2 may be variable (Fig. 3b)^92^. The sequence specificity of each caspase has been determined based on peptide-bound experimental structures that do not consider the full-length protein substrate scaffold. Hence, defining the functional role of discrete proteolytic events within the global substrate pool is a major biological objective. Future studies will be needed to improve our knowledge of caspase substrate recognition beyond the P4–P1 substrate sites.

## Caspase substrate cleavage

Several cellular and proteomic approaches have been used to identify how specific caspases cleave their substrates after an aspartic acid in different innate immune and cell death pathways^67,181^. For instance, the executioner caspase-3 can specifically cleave human GSDME at the consensus motif DMPD, allowing pore formation and eventual cell death^72,99^. Mutations in either Asp residue within the DMPD peptide motif confer resistance to cleavage^72^. This underscores the critical role of this motif in the activation of GSDME by caspase-3. While the other executioner, caspase-7, shares the same recognition motif (DXXD) as caspase-3^184,185^, it cannot cleave GSDME^72,186^. This distinction suggests a unique activation mechanism for GSDME by caspase-3. Moreover, although caspase-3 and -7 can cleave specific substrates such as poly (ADP ribose) polymerase-1 (PARP1), RhoGDI, and ROCK 1 with similar efficiency, these proteases display differences in their ability to cleave many other substrates, with caspase-3 having a much broader array of substrate specificity than caspase-7^187^. This may be because caspase-3 has extremely high *K*_*cat*_ values, allowing it to process substrates that are considered specific for other caspases^188,189^.

Additionally, studies using subtiligase reverse N-terminomics have shown that caspase-1 cleaves its substrate GSDMD at the FLTD tetrapeptide motif^181,183^. In vitro experiments confirmed that the cleavage of GSDMD by caspase-1 occurs rapidly at 1.3 × 10^5^ M/s (*K*_*cat*_*/K*_*M*_). Caspase-1-mediated cleavage of GSDMD releases its N-terminal domain to facilitate pore formation and subsequent lytic cell death^19,20,66,162,163^. Caspase-1 was originally identified as the processing enzyme for pro-IL-1β and IL-18^190–193^, and mature IL-1β and IL-18 are released through the GSDMD pores^18–20^^,194^^,195^. Studies have shown that caspase-4 and -5 also play a role in the maturation of IL-18 in vitro and during bacterial infections^63,196,197^, and the tetrapeptide substrate specificity for caspase-1 and -4 is the same in recognizing IL-18^196^. Furthermore, comparisons of structure and sequence identified that caspase-5 contains the conserved residues that mediate caspase-4 binding to IL-18^196^. However, the murine homolog of caspase-4/-5, caspase-11, is unable to cleave IL-18. Mutations that replace caspase-11 active site and/or exosite residues with those from caspase-4 restore the cleavage ability^196^. Together, these structure–function relationships define the role of multiple caspases in innate immune responses.

## Role of caspase exosites in substrate recognition

Improved structural understanding of the caspases has shown that the caspase active site is not the only determinant of the substrate tetrapeptide sequence that is cleaved. A secondary binding site (exosite) distinct from their active site has also been identified^198,199^. An exosite in caspase-7 was found to facilitate the binding and cleavage of PARP1 and heat shock protein 90 (Hsp90). The lysine residues (K(38)KKK) in the N-terminal region of caspase-7 are critical for the proteolysis of these substrates. Although caspase-3 and -7 share similar tetrapeptide specificity, caspase-7 is more effective in cleaving these substrates because of its secondary binding site^200^. Structures of caspase-1, -4, and -11 in complex with the GSDMD C-terminal domain (GSDMD-CTD) show that the GSDMD-CTD plays a role in the recognition of these inflammatory caspases through a small β-sheet, referred to as an exosite^201^. These findings suggest that the tertiary structure of GSDMD is critical for its recognition by inflammatory caspases and the subsequent activation of inflammatory cell death^201^. However, these structures do not explain how inflammatory caspases can simultaneously recognize full-length GSDMD through exosites and active sites. Recently, a crystal structure of full-length human caspase-1 complexed with murine GSDMD showed an exosite situated 20 Å away from the caspase-1 active site (Fig. 3c)^198^. Structural and mutational analyses of the GSDMD-CTD suggest that the GSDMD-CTD (exosite region) serves as a caspase recruitment module, in addition to its role as an autoinhibition domain that regulates the cytolytic function of the GSDMD-N-terminal domain (NTD). Caspase-1 engages with GSDMD through dual interfaces, which is crucial for substrate selectivity and limits the number of physiological substrates^198,201^. In addition, the crystal structure of caspase-4 complexed with pro–IL-18 has shown a dual site substrate recognition, and a similar dual substrate recognition mechanism is observed in caspase-5 and caspase-1 to process pro-IL-18^196^. In mouse caspase-11, structural divergence was observed around the exosite region, resulting in its inability to process pro-IL-18. The structure of pro-IL-18 takes an autoinhibitory conformation due to the interactions between the pro-peptide and the post-cleavage site region, which prevents recognition by IL-18Rα. The cleavage of pro-IL-18 by caspase-1, -4, or -5 results in significant conformational changes, creating two critical receptor-binding sites for the matured IL-18^196,202^. While these findings demonstrate the importance of the exosites in caspase-mediated proteolysis of their substrates, quantitative analyses will be needed to demonstrate the dominant role of an exosite in its initial enzyme–substrate association. These findings highlight the importance of studying full-length caspases with their respective substrates to provide novel insights into structure-based drug design.

## Caspase pro-domains

While caspase active sites and exosites drive substrate recognition and cleavage, caspase pro-domains are central to their protein–protein interactions, making them critical for caspase function and regulation. The caspase pro-domains (i.e., CARD and DED) belong to the death domain (DD) superfamily and facilitate the formation of oligomeric supramolecular complexes via self-association or interaction with other CARD/DED-containing proteins^167,168,203,204^. The number of amino acids in the pro-domain varies between 119 and 219, and these domains possess specialized sequences that interact with their activation scaffolds. Caspase-8 and caspase-10 contain two DEDs that are known to interact with similar domains on adaptor proteins, while caspase-1, -2, -4, -5, and -9 contain CARDs that interact with the CARDs of their respective adaptor or effector proteins. Caspase-11 and -12 also contain CARDs, but these are less well characterized. In contrast, caspase-3, -6, -7, and -14 have short or no pro-domains, without CARD or DED regions (Fig. 1)^92^.

The pro-domains are critical for a variety of protein–protein interactions in cell death complex formation. For instance, inflammasomes recruit pro-caspase-1 through a CARD–CARD interaction, leading to the activation of caspase-1 to drive inflammatory cell death^2,14^. Similarly, PANoptosomes contain multiple caspases. For example, during IAV infection, PANoptosis is associated with the activation of multiple caspases, including caspase-1, caspase-8, caspase-3, and caspase-7, and is further augmented by caspase-6^21–27,74^. Microscopy and co-immunoprecipitation studies have shown that multiple caspases, including caspase-1 and -8, are key components of PANoptosomes^21–26,75^. The formation of the PANoptosome has been hypothesized to involve CARD–CARD, PYD–PYD, and PYD–DED interactions^6,95^, but their specific molecular interactions remain understudied. Understanding the mechanisms of DED or CARD interactions with adaptor proteins is an area of intensive study that has been investigated and reviewed elsewhere^167,168,203,205^. Structural and sequence analyses of the pro-domains of caspases have identified a limited sequence similarity, but high structural similarity^87^, suggesting a critical role for caspase structure in their functions.

### Caspase-CARD interactions

The CARD is a well-known protein interaction module in the DD superfamily. CARDs are key death domain folds that have evolved to be part of innate immune sensors or death receptors and often mediate the formation of supramolecular signaling complexes to induce caspase activation and downstream signaling. CARDs can be found in caspase-1, -2, -4, -5, -9, -11, -12, and common CARD-containing proteins for interaction are ASC, APAF-1, CARMA1, BCL10, NOD2, NLRC4, MAVS, and RIG-I^206^. The CARDs interact in a homotypic or heterotypic manner with their partners, and disruption of these interactions has been considered a therapeutic target for infectious and inflammatory diseases as well as cancers^11,166,167^. The known structures and sequences of CARD-containing proteins show that they have low sequence similarity but share a similar structural fold. The hallmark of the CARD-containing proteins is their 6 helical bundles, which is a common structural feature of the DD superfamily^167^. Caspases containing a CARD pro-domain at the N-terminal region are required for the assembly of supramolecular complexes in the signaling cascade. For example, APAF-1 CARD interacts with the caspase-9 CARD during apoptosome formation. The binding surface of APAF-1 CARD is primarily acidic and involves interactions among the α2, α3, and α5 helices. The α2, α3, and α5 helices interact with α1 and α4 of the corresponding partner CARD, forming an antiparallel four-helix bundle. It has been suggested that the α5 helical motif undergoes a binding-induced conformational change, promoting the complex formation^207^.

**Fig. 4:**
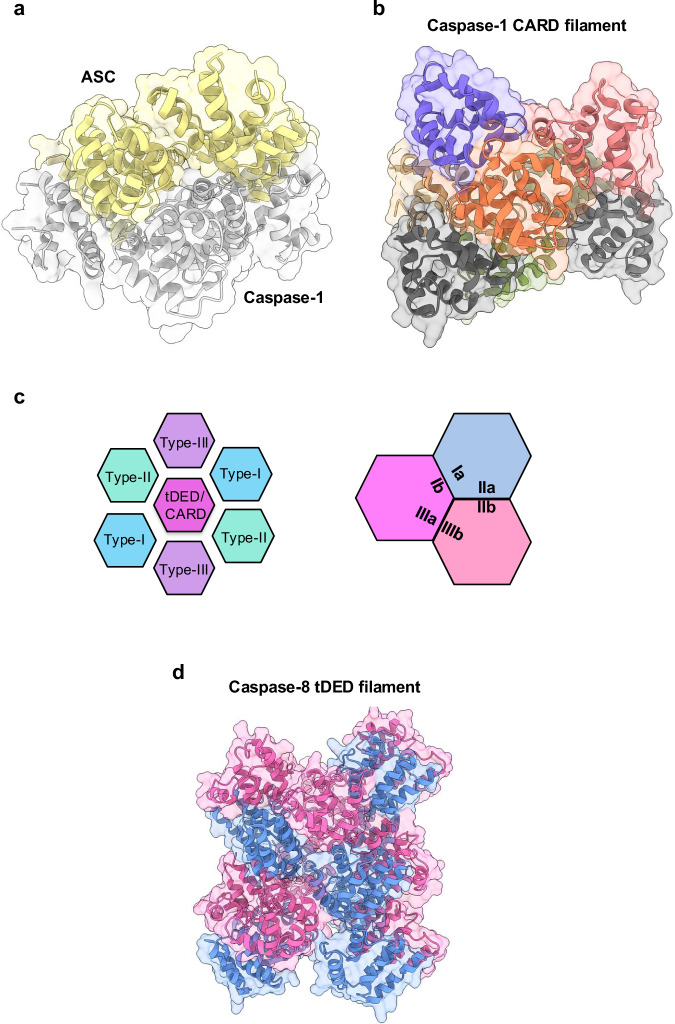
Structural analysis of caspase pro-domain and interactions with its partners. **a** Illustration of ASC:caspase-1 CARD octamer complex structure (PDB ID: 7KEU); ASC is shown in yellow, and caspase-1 in gray. **b** Representation of the caspase-1 CARD filament (PDB ID: 5FNA); each individual monomer unit of caspase-1 CARD is represented by a different color for clarity. **c** Illustration of the three common types of interactions between tadem DED (tDED) or CARD monomers of the caspases. **d** Structural representation of caspase-8 tDED filament (PDB ID: 5L08); the individual monomer tDED molecules are colored in pink and cornflower blue, respectively, to indicate the formation of the tDED filament.

Similarly, the CARD of ASC interacts with the caspase-1 CARD and forms the inflammasome (Fig. 4a)^14,208^. Over the last two decades, extensive research has been conducted on the molecular components and activation mechanisms of different inflammasomes, which have been implicated in several infectious and inflammatory diseases and cancers^209^. Given its critical role in inducing cell death, as well as disease relevance, understanding the mechanisms of inflammasome formation is a central research focus. Cryogenic electron microscopy (cryo-EM) structures of homodimer/heterodimer CARD filaments have provided structural insights into the nucleation and polymerization of these higher-order assemblies (Fig. 4b)^168^. Filamentous structures of ASC and NLRC4 CARDs indicate that they both share similar helical assembly patterns, with type-I & II interfaces forming between interstrand interactions and type-III interfaces displaying intrastrand interactions; together, these interfaces are composed of strong charge–charge interactions (Fig. 4c). Based on these observations, it is possible that the downstream caspase-1 CARD recruitment occurs through charge complementarity^168^.

Inflammasomes can also be integral components of larger multiprotein, cell death-inducing complexes called PANoptosomes to drive PANoptosis^21–26^^,74^. Several studies have suggested potential roles for CARDs in PANoptosis. For example, the filamentous CARD-containing adapter ASC is a core component of many PANoptosomes and could recruit caspase-1 to allow PANoptosomes to efficiently process proinflammatory cytokines, such as IL-1β and IL-18^21–26^. However, further biochemical analyses, as well as cryo-EM or cryogenic electron tomography (cryo-ET), will be useful to demonstrate the involvement of caspase–CARD interactions in the formation of PANoptosomes.

### Caspase-DED and heterotypic interactions

Activation of membrane-bound death receptors following ligand binding induces receptor multimerization followed by conformational changes of the intracellular DED to recruit adaptor proteins via homotypic DD interactions^203,205^. The DED-containing adaptor protein may then recruit its partner caspases through similar DED/DED interactions^210^. In addition to caspase-8 and -10, DED-containing proteins that can interact with caspases also include FADD, c-FLIP, PEA-15, DEDD, and DEDD2^206^. The importance of DED/DED interactions in innate immunity is well documented. For example, FADD recruits its downstream partner caspase-8/-10 in response to its intracellular activation^211^. FADD and caspase-8 are key components of the canonical CD95, TRAIL-R, TNFR complex-II, and RIPK1/RIPK3-containing ripoptosome complexes^49,212^, as well as the cytosolic FADDosome complex^213^. DED-mediated interactions between FADD and caspase-8 can drive cell death in these scenarios. In addition, recent studies suggest that the lytic cell death adaptor protein ASC recruits caspase-8 as a binding partner via PYD/tandem DED (tDED) interactions to regulate cell death^214,215^. In addition, regulation of the inflammasome can depend on caspase-8 and FADD, and these interactions may be critical for this regulation^69^. The first structure of the caspase-8 tDED in its filament form identified three types of interactions for asymmetric interfaces, similar to CARD filaments (Fig. 4d)^205^.

Beyond the inflammasome, the caspase-8 DED is also a critical component for the formation of other multiprotein complexes. For instance, in the assembly of death receptor signaling complexes, homotypic interactions between the pro-caspase-8 tDEDs and DED of FADD are critical^205,216^. Additionally, the caspase pro-domain homotypic/heterotypic interactions between proteins facilitate PANoptosome formation. Heterotypic interactions between the PYD of the central PANoptosome component ASC and the DED of caspase-8^215^ are likely essential to bring together the components of the PANoptosome. In addition, caspase-6 is critical to facilitate complex formation in the context of the ZBP1-PANoptosome, where caspase-6 potentiates the interaction between ZBP1 and RIPK3 to drive inflammatory cell death^26^; this likely occurs through heterotypic interactions and is independent of caspase-6’s enzymatic activity. Co-immunoprecipitation studies with full-length ZBP1, RIPK3, and inactive caspase-6 showed enhanced binding of ZBP1 to RIPK3^26^. Furthermore, in vitro studies with truncated domains of each of these proteins suggest that both the N-terminal and C-terminal domains of caspase-6 interact with N- and C-terminal regions of RIPK3, possibly via intrinsically disordered regions (IDRs). Together, these findings suggest that caspase-6 IDRs enable the assembly of the ZBP1 and RIPK3 complex in the ZBP1-PANoptosome^26^. However, to establish the comprehensive mechanism of how these supramolecular structures assemble and recruit their respective caspases, determination of the full-length structures of these protein complexes will be needed. The structural information may provide precise insights into the conformational selection mechanism of each partner during their assembly and identify the exact heterodimer interfaces between them to inform structure-based drug design.

## Caspases in disease and therapy

Given the diverse roles of caspases, genetic mutations in caspases can affect the onset and outcome of many diseases, including susceptibility to infection, inflammatory diseases, cancer, neurodegenerative diseases, and many others^11,93^. Defects in caspase activation can promote tumorigenesis or infection, while excessive caspase activation can lead to aberrant cell death and inflammation to promote neurodegeneration, cancer, or inflammatory diseases. For example, emerging evidence suggests that excessive caspase-6 activation contributes to axon degeneration in Alzheimer’s and Huntington’s disease^217–221^, though this remains controversial. Mutations that alter caspase function have been identified in several cancers^34^^,^^222–224,225–231^. For example, chain terminator R68* and Q97* mutations in the N-terminal region of pro-caspase-8 abolish TRAIL-induced dimerization and its ability to trigger apoptosis; these mutations are associated with the development of head and neck squamous cell carcinoma (HNSCC) tumors^225^. Other truncation mutants that retain both DEDs, such as S375* and S386*, have also been found. These mutants may inhibit caspase-8 activation through the formation of non-functional dimers^225^, potentially predisposing patients to cancer development. In addition, the L105H mutation has been identified in patients with HNSCC. This mutation provides resistance to cell death, which may contribute to worse outcomes in patients with HNSCC^225,226^. However, the details of the specific inhibitory mechanism of the L105H missense mutation on caspase-8 activation remain unclear. Furthermore, patients with acute myeloid leukemia with the pro-caspase-8 mutation P10 or the Q428H variant are more likely to resist chemotherapy^227^. A specific missense mutation (C70Y) has also been identified in the p23 large subunit of caspase-7, which may result in a loss of cell death function in vivo, and this inactivating mutation may contribute to the pathogenesis of some human solid cancers^228^. However, because caspase-7 can be activated downstream of BAX/BAK-dependent mitochondrial permeabilization^43,232^, and this permeabilization can drive caspase-independent cell death^233^, the role of this mutation in the pathogenesis of human solid cancers remains speculative. Additionally, the Q221R variant in caspase-9 has been suggested to induce a conformational change that might affect the interaction with APAF-1 and potentially contribute to colon cancer^229,230^. However, APAF-1 is not a tumor suppressor gene, and APAF-1-null cells still undergo cell death in response to cytotoxic stimuli^112^. Therefore, the functional consequences of the Q221R mutation on caspase-9 activity and its potential role in cancer development require further experimental validation. Moreover, caspase-5 mutations, including frameshift, missense, and silent mutations, have been found across diverse cancer types, with a notable incidence in gastric carcinomas^231^. Caspase-1 and caspase-4 mutations were also identified, involving a missense mutation resulting in amino acid substitution^231^ (Table 1). These findings highlight the critical roles of caspases in tumor development and provide insights into potential mechanisms of inhibition and loss of function caused by these mutations.

Furthermore, genetic mutations in components of caspase signaling pathways can induce dysregulated caspase activation and are also clinically significant^34^. Gain-of-function mutations have been identified in innate immune PRR sensors that activate caspases. For instance, gain-of-function mutations in NLRP1 increase susceptibility to skin inflammation and skin cancer^234^. Primary keratinocytes from these patients produce increased levels of IL-1β, leading to spontaneous inflammation^235,236^. Moreover, NLRP1 mutations that lead to elevated activation of caspase-1 and IL-18 have been found in patients who have skin dyskeratosis, arthritis, and periodic fever^237^. In addition, gain-of-function mutations in NLRP3 have been identified as the cause of cryopyrin-associated periodic fever syndromes (CAPS)^238–240^. CAPS is a collective term that includes three autosomal dominant disorders, namely familial cold autoinflammatory syndrome (FCAS), Muckle-Wells syndrome (MWS), and neonatal-onset multisystem inflammatory disease (NOMID). Studies conducted using an FCAS mouse model have identified that caspase-1-mediated cell death, as well as the release of IL-1β, IL-18, and TNF, contribute to the pathophysiology^241–243^.

In addition to their role in cell death pathways and inflammation, caspases are also involved in various other cellular processes that are linked to disease. In mammals, caspase-3 inhibitors (CI3, C3I4), in combination with caspase-9 inhibitors (C9I2), can prevent axon regeneration in dorsal root sensory neurons by preventing the formation of the growth cone^244^. Calpain, a calcium-activated protease, may activate caspase-3 during this process^245^. Caspase-3 has also been identified as a regulator of spine density and dendrite morphology, and deficiency in this enzyme is associated with extra spines. Caspase-2 plays a role in controlling dendritic spine density and cognitive function^246^. Moreover, caspases, such as caspase-9 and caspase-3, are involved in axonal guidance and synaptogenesis, which affect the proper projection of axons and the formation of synapses. Additionally, caspase-3 is linked to actin cytoskeletal rearrangement during both apoptotic and non-apoptotic processes, such as macrophage polarity formation^247^.

Several caspase inhibitors, both natural and synthetic, have been developed for potential therapeutic applications in multiple pathologies (Table 1). A serine protease inhibitor cytokine response modifier A (CrmA) was the first natural inhibitor discovered to act on caspase-1, -8, and -10, and it can efficiently reduce inflammation by preventing cell death and the production of IL-1β and interferon γ^248–250^. In addition, several synthetic caspase inhibitors have also been developed as potential treatments for diseases associated with aberrant cell death. Typically, these inhibitors are categorized as peptide-based and non-peptide-based compounds^251^. One of the peptide-based inhibitors, Belnacasan, is a reversible inhibitor of inflammatory caspases that blocks the active site of caspase-1 to prevent inflammation in animal models^252^. Emricasan, the first FDA-approved irreversible broad-spectrum caspase inhibitor, has anti-cell death and anti-inflammatory effects, and it is currently undergoing broad clinical testing. The selective nature of this pan-caspase inhibitor has prompted research to focus on its potential applications^253,254^. Another irreversible pan-caspase inhibitor, VX-166, reduces hepatocellular cell death, inflammation, and fibrosis in models of nonalcoholic steatohepatitis (NASH) induced by a methionine-choline diet (MCD) or high-fat diet (HFD)^255^. Similarly, M867, a peptidomimetic caspase-3 inhibitor, reduces tumor progression and vasculature in H460 lung cancer cells in vitro and in vivo in response to ionizing radiation^256^. The other additional caspase inhibitors and their clinical use have been previously reviewed elsewhere^251,257^ (Table 1).

While multiple caspase inhibitors are being developed for therapeutic applications, only a limited number of compounds have reached clinical trials due to a lack of specificity and efficacy, toxicity issues, and the development of drug resistance^251^. The recent ability to develop nanobodies targeting other cell death molecules, such as GSDMD or ASC^258,259^, suggests a potential to develop new, selective caspase inhibitors through similar techniques. To continue improving the design of effective caspase inhibitors for different diseases, it remains critical to improve our understanding of the structure and functions of multiple caspases in disease models.

## Summary and future perspectives

Caspases are central regulators of cell death and inflammation as part of the innate immune system; hence, their signaling pathways are attractive targets for structure-based drug design to mitigate pathological responses in various infectious and inflammatory diseases and block cancer progression. The structure of caspase-1 (PDB ID: 1ICE) was the first to be solved in 1994^260,261^, and now approximately 329 human caspase structures have been deposited in PDB. These structures, combined with rapid progress in functional studies, have led to the design of small molecules to target different caspases, as well as improved understanding of their functional impacts across in vitro and in vivo preclinical studies. Over the past decade, tremendous progress has been made in caspase biology, including in identifying the crosstalk among different caspases and the identification of the role of caspases in driving inflammatory cell death. This progress has elucidated significant molecular connections among apoptosis, pyroptosis, and necroptosis, which could not be addressed by these established cell death models, leading to the identification of PANoptosis^6,9^. For instance, caspase-1, an essential pyroptotic molecule, cleaves caspase-7 during *Salmonella* infection and in response to LPS plus ATP stimulation^67,68^. Caspase-1 has also been shown to cleave the apoptotic PARP1 in response to inflammation-activating triggers^68^, and the loss of caspase-1 or GSDMD leads to the activation of caspase-3, -7, -8, or -9^262–264^. Furthermore, caspase-8 can regulate pathways that activate caspase-1^69^, and caspase-1 and -8 can have functionally redundant roles in disease contexts^70^. Additionally, caspase-8 has been well-established as a regulator of apoptosis, and emerging evidence establishes its critical role in PANoptosis, acting as a core component of the PANoptosome^21–26,74,75^. Given caspase-8’s multifaceted regulatory roles in both non-lytic and lytic cell death, any impairment in its function can lead to several human diseases and solid tumors^37,60,69,108,265^. Up to 10% of human HNSCC tumors, as well as uterine, stomach, cervical, colorectal, and bladder cancers, can be attributed to the loss of caspase-8 or mutations in caspase-8^37,60,266,267^. Additionally, advances in caspase research have led to the identification of the non-canonical inflammasome and the role of different GSDMs as cell death executioners^18–20,66,72,79,98,99,162,163,268^. Collectively, these advances have provided novel targets for therapeutic applications that are rapidly progressing through preclinical studies. It is likely that caspases have further impacts on cancers and diseases, which are not yet fully understood. Therefore, it would be beneficial to improve our understanding of the molecular mechanism and structural impacts of caspases and their roles in supramolecular protein complexes, such as PANoptosomes. This understanding is crucial for the development of new therapeutics.

It will also be critical to further understand the non-enzymatic functions of caspases to define their biological mechanisms. Caspases can use homotypic interactions among DEDs and CARDs to form a scaffold for oligomerization, which can induce proximity-dependent activation of caspases or the caspase activity-independent assembly of oligomeric signaling scaffolds. For example, caspase-8 scaffolds play a critical role in the assembly of a caspase-8-FADD-RIPK1 complex in a protease-independent manner^213^. Additionally, the executioner caspase-6 regulates ZBP1-mediated PANoptosome formation and PANoptosis in a non-enzymatic manner^26^. Much of this knowledge has been gained through cell death phenotypes and genetic studies to identify functional roles of caspases. To better understand how caspases drive innate immunity and how they can be targeted for therapeutic purposes, it is essential to investigate the intricacies of innate immune cell death complexes and their interactions with caspases and other cell death executioners at the molecular and structural levels. Advanced techniques such as cryo-ET, combined with detailed proteomic and molecular biology approaches, are well-positioned to address these questions and open new avenues to use structural immunology to determine caspase functions in health and disease and inform therapeutic strategies.
